# Anticipated Survival and Health Behaviours in Older English Adults: Cross Sectional and Longitudinal Analysis of the English Longitudinal Study of Ageing

**DOI:** 10.1371/journal.pone.0118782

**Published:** 2015-03-23

**Authors:** Jean Adams, Elaine Stamp, Daniel Nettle, Eugene M. G. Milne, Carol Jagger

**Affiliations:** 1 Institute of Health & Society, Newcastle University, Newcastle upon Tyne, United Kingdom; 2 Centre for Behaviour & Evolution, Institute of Neuroscience, Newcastle University, Newcastle upon Tyne, United Kingdom; 3 Institute of Ageing & Health, Newcastle University, Newcastle upon Tyne, United Kingdom; University of Glasgow, UNITED KINGDOM

## Abstract

**Background:**

Individuals may make a rational decision not to engage in healthy behaviours based on their assessment of the benefits of such behaviours to them, compared to other uncontrollable threats to their health. Anticipated survival is one marker of perceived uncontrollable threats to health. We hypothesised that greater anticipated survival: a) is cross-sectionally associated with healthier patterns of behaviours; b) increases the probability that behaviours will be healthier at follow up than at baseline; and c) decreases the probability that behaviours will be ‘less healthy’ at follow than at baseline.

**Methods:**

Data from waves 1 and 5 of the English Longitudinal Survey of Ageing provided 8 years of follow up. Perceptions of uncontrollable threats to health at baseline were measured using anticipated survival. Health behaviours considered were self-reported cigarette smoking, physical activity level, and frequency of alcohol consumption. A wide range of socio-economic, demographic, and health variables were adjusted for.

**Results:**

Greater anticipated survival was cross-sectionally associated with lower likelihood of smoking, and higher physical activity levels, but was not associated with alcohol consumption. Lower anticipated survival was associated with decreased probability of adopting healthier patterns of physical activity, and increased probability of becoming a smoker at follow up. There were no associations between anticipated survival and change in alcohol consumption.

**Conclusions:**

Our hypotheses were partially confirmed, though associations were inconsistent across behaviours and absent for alcohol consumption. Individual assessments of uncontrollable threats to health may be an important determinant of smoking and physical activity.

## Introduction

Poor engagement in health-promoting behaviours is an important determinant of morbidity and mortality and results in substantial social, healthcare and economic costs worldwide. In the UK, individuals who smoke, do not engage in regular physical activity and do not consume moderate levels of alcohol and a healthy diet, are 3.5 times more likely to die over 20-years than those who do.[1] The costs of these behaviours to the UK health care system have been estimated to be £13.3bn per annum.[2] Recent figures from England indicate that one fifth of adults are current smokers, one third consume more than recommended levels of alcohol,[3] two-thirds do not meet recommended physical activity levels[4] and almost three-quarters consume less than the recommended intake fruit and vegetables.[5]

Unhealthy behaviours are the result of a combination of environmental constraints making some behaviours practically difficult to adopt and maintain,[6] social norms making some behaviours socially unacceptable,[7] lack of knowledge of why behaviour should be changed, and lack of skills to enable change.[8] However, all of these explanations rest on an assumption that individuals would adopt healthy behaviours if they understood the benefits, and lived in environments that facilitated adoption.[9]

An additional explanation of poor engagement with healthy behaviours is that individuals make a rational decision not to engage, based on their assessment of the benefits to them. Thus, it is suggested that individuals weigh up the likely benefits of any particular health-promoting behaviour against their assessment of the other, uncontrollable, risks to their health to which they are exposed. Those who believe they are exposed to substantial uncontrollable risks to their health may make a rational judgement that the additional benefits of adopting any particular health-promoting behaviour is likely to be minimal. In contrast, those who believe that such uncontrollable risks are minimal will weigh the relative benefit of healthy behaviours as greater.[10, 11]

We are aware of only one previous study exploring the association between perceived risks to health and health behaviours.[12] This cross-sectional study, using a convenience sample, found that lower perceived levels of uncontrollable risks to health were associated with greater reported efforts to improve health.

Here we use individuals’ assessments of their estimated remaining lifespan (‘anticipated survival’) as a marker of subjective judgements of uncontrollable risks to health given their current circumstances. This is a less nuanced marker of uncontrollable risks to health as than used previously—where the difference between anticipated survival given no personal effort to protect health, and maximal possible personal effort to protect health was used. We discuss the implications of this in the Discussion section.[12] We explore cross-sectional associations between anticipated survival and health behaviours in a population-representative cohort of older English adults. We hypothesised that greater anticipated survival is cross-sectionally associated with healthier behaviours.

A cross-sectional association between anticipated survival and health behaviours is open to the possibility of ‘reverse-causation’—where healthy behaviours result in longer anticipated survival, in addition to, or instead of, longer anticipated survival resulting in healthy behaviours. To reduce the possibility of this, we explore the longitudinal relationship between anticipated survival and change in health behaviours over eight years of follow up. We hypothesised that greater anticipated survival increases the probability that behaviours will be ‘healthier’ and decreases the probability that behaviours will become ‘less healthy’ over time.

## Methods

### Data source and inclusion criteria

The English Longitudinal Survey of Ageing (ELSA) is a prospective cohort study designed to be representative of individuals aged 50 or older living in private households in England.[13, 14] Data from waves 1 and 5, collected in 2002–03 and 2010–11, were used. All core ELSA participants who took part in full interviews, in person, during both waves 1 and 5 were eligible for inclusion in the analyses.

### Variables of interest

#### Anticipated survival

Anticipated survival was measured using the question: “What are the chances (from 0–100) that you will live to be x years or more?” For individuals aged ≤65 years, x = 75; for those aged 66–69 years, x = 80; for those aged 70–74 years, x = 85; for those aged 75–79 years, x = 90; for those aged 80–84 years, x = 95; for those aged 85–99 years, x = 100. Due to response spikes at 25%, 50%, and 75%, responses were grouped into tertiles for analysis: low (0–33% chance of survival), moderate (33–66%), and high (66–100%). Differences in the target age that individuals were asked about can be taken into account by controlling for the difference between current and target age. As this did not change any results, we report results without this control.

#### Health behaviours

Cigarette smoking at waves 1 and 5 was determined using the question “Do you smoke cigarettes at all nowadays?”, with responses of yes or no. Total physical activity was coded as sedentary, low, moderate or high using answers to questions on occupational activity, sports and other activity.[15]

At wave 1, alcohol consumption was determined using the question: “In the past 12 months have you taken an alcohol drink….”, with response options of not at all, on special occasions only, once or twice a month, once or twice a week, daily or almost daily, and twice a day or more. At wave 5, this question was reworded to: “How often have you had an alcoholic drink of any kind in the last 12 months?”, with response options of not at all, once or twice a year, every couple of months, once or twice a month, once or twice a week, three or four times a week, five or six times a week, and almost every day. These two coding systems were harmonised into: not at all; less than once a month; once or twice a month; once or twice a week; and more than twice a week.

For brevity, we use the term ‘healthy’ to describe not smoking, being more physically active or consuming alcohol less frequently; and ‘unhealthy’ to describe alternate behaviours.

#### Demographic, health and socio-economic variables

A range of potential confounding factors were included that have previously been reported to be associated with anticipated survival, health behaviours, or both.

Self-reported gender was coded as male or female. Age at wave 1 was grouped into six categories, 50–65, 66–69, 70–74, 75–79, 80–84, or 85 years and older, corresponding to the bands used in the question on anticipated survival. Marital status was coded as never married, currently married, divorced or separated and not remarried, or widowed and not remarried.

Activities of daily living (ADL) score was calculated from the number of difficulties due to health problems in dressing, walking across a room, bathing or showering, eating, getting out of bed, and using the toilet, (score 0–7). Instrumental ADL (IADL) score was calculated from the number of difficulties due to health problems with using a map, preparing a hot meal, grocery shopping, making telephone calls, taking medications, doing housework or gardening, and managing money (score 0–7).[16] Both ADL and IADL scores were collapsed into 0, 1–2, or 3+ difficulties for analysis.

Self-rated health was assessed using the question “Would you say your health is…” with response options of excellent, very good, good, fair or poor. These were collapsed into excellent or very good, good, and fair or poor for analysis. The presence of a long-term illness was assessed with yes or no answers to the question “Do you have any longstanding illness, disability or infirmity?” We did not additional adjust for limiting long-term illness as limitation is more adequately captured using the ADL and IADL scores described above.

Depression was measured using the CES-D eight-item scale that has been widely used as a screening tool.[17, 18] As previously,[19] scores were dichotomised into those that did (4–20) and did not (0–3) suggest clinically meaningful depression.

Socio-economic position was measured using the Index of Multiple Deprivation score (IMD) of current area of residence.[20] Scores were categorised as least (deciles 1–3), moderately (deciles 4–6), and most deprived (deciles 7–10) for analyses.

#### Analysis

Cross-sectional associations between health behaviours and anticipated survival at wave 1, before and after adjusting for relevant potential confounders measured at wave 1, were explored using ordinal logistic regression. Separate models were run for each behaviour, with behaviours as outcome variables and anticipated survival, and potential confounders, included as explanatory variables. In these models behaviours are coded with higher scores representing ‘more’ of each behaviour (i.e. smoking, being more physically active or consuming alcohol more frequently).

To determine the association between anticipated survival and change in health behaviours, we first calculated change variables for each behaviour in three categories: change to less healthy behaviour, no change, and change to more healthy behaviour. Multinomial logistic regression was used to explore the relationship between anticipated survival at wave 1 and change variables, adjusted for baseline behaviour throughout. As before, separate models were run for each behaviour.

In both groups of models, potential interactions between anticipated survival and other explanatory variables were explored, but none were found. Most analyses were conducted on an available case basis in Stata v11.0. However, unadjusted and adjusted versions of the ‘same’ models were nested within each other.

#### Ethics statement

Ethical approval for all the ELSA waves was granted from the National Research and Ethics Committee. Participants gave full informed written consent to participate in the study. Separate ethical approval for this secondary analysis of anonymised data was not required.

#### Data sharing

We obtained data for these analysis from the UK Data Archive. Conditions of data use from the UK Data Archive include that it is not shared beyond the registered user. However, other eligible registered users can obtain the same data from the UK Data Archive.

## Results

A total of 6242 individuals were eligible for inclusion in the analysis. The distribution of anticipated survival, demographic, health and socioeconomic variables at wave 1 are shown in Table 1. Around half of participants reported moderate anticipated survival, with low being the least common option. Most participants were 65 years or younger, currently married, had ADL and IADL scores of zero and did not have a CES-D score indicative of clinically meaningful depression. Around half of participants reported having a longstanding illness, and half reported their health as excellent or very good. Participants were less likely to live in more deprived areas than the English population as a whole.

**Table 1:** Distribution of anticipated survival and other participant characteristics at wave 1, English Longitudinal Study of Ageing.

Baseline variable	Level	Frequency, n (%)
Anticipated survival	Low	761 (12.6)
	Moderate	3101 (51.3)
	High	2189 (36.2)
Sex	Male	2686 (43.8)
	Female	3442 (56.2)
Age (years)	50–65	3901 (63.7)
	66–69	789 (12.9)
	70–74	717 (11.7)
	75–79	443 (7.2)
	80–84	229 (3.7)
	85+	49 (0.8)
Marital Status	Never married	303 (5.0)
	Currently married	4247 (69.3)
	Divorced/separated	705 (11.5)
	Widowed	872 (14.2)
ADL^1^ score	0	4909 (80.1)
	1–2	880 (14.4)
	3+	339 (5.5)
IADL^2^ score	0	5150 (84.0)
	1–2	809 (13.2)
	3+	169 (2.8)
CES-D^3^ score	<4	5209 (85.9)
	≥4	858 (14.1)
Self-reported health	Excellent or very good	2894 (47.3)
	Good	1937 (31.6)
	Fair or poor	1294 (21.1)
Long term illness, disability or infirmity	No	2883 (47.0)
	Yes	3244 (53.0)
IMD^4^	Least deprived	2334 (38.1)
	Moderately deprived	2013 (32.9)
	Most deprived	1781 (29.1)

^1^ADL: Activities of Daily Living;

^2^IADL: Instrumental Activities of Daily Living;

^3^CES-D: Centre for Epidemiologic Studies Depression Scale;

^4^IMD: Index of Multiple Deprivation

Table 2 shows the distribution of health behaviours at waves 1 and 5 overall and by anticipated survival at wave 1. At both points, most participants were non-smokers, drank once or twice a week or more frequently, and took part in moderate or high levels of physical activity. Between waves 1 and 5, non-smoking became more common, physical activity levels decreased, and both not consuming alcohol and consuming it more than once or twice a week became more common.

**Table 2:** Distribution of health behaviours at waves 1 and 5 by anticipated survival at wave 1, English Longitudinal Study of Ageing.

		Wave 1				Wave 5			
Behaviour	Category	Low AS^1^, n(%)	Moderate AS, n(%)	High AS, n(%)	Total, n(%)	Low AS, n(%)	Moderate AS, n(%)	High AS, n(%)	Total, n(%)
Cigarette smoking	Non-smoker	613 (80.6)	2555 (82.4)	1889 (86.3)	5057 (83.6)	648 (85.2)	2731 (88.1)	1988 (90.9)	5367 (88.7)
	Smoker	148 (19.5)	546 (17.6)	300 (13.7)	994 (16.4)	113 (14.9)	368 (11.9)	200 (9.1)	681 (11.3)
Physical activity	Sedentary	86 (11.3)	141 (4.6)	70 (3.2)	297 (4.9)	195 (25.7)	293 (9.5)	152 (7.0)	640 (10.6)
	Low	229 (30.1)	633 (20.5)	364 (16.6)	1226 (20.3)	265 (34.9)	826 (26.7)	471 (21.6)	1562 (25.9)
	Moderate	350 (46.0)	1696 (54.8)	1146 (52.4)	3192 (52.8)	244 (32.1)	1479 (47.8)	1125 (51.6)	2848 (47.2)
	High	96 (12.6)	625 (20.2)	609 (27.8)	1330 (22.0)	56 (7.4)	494 (16.0)	434 (19.9)	984 (16.3)
Alcohol consumption	Not at all	99 (13.0)	266 (8.6)	171 (7.8)	536 (8.9)	119 (20.7)	380 (13.9)	214 (10.9)	713 (13.5)
	<Once per month	171 (22.5)	551 (17.8)	324 (14.8)	1046 (17.3)	130 (22.6)	470 (17.2)	311 (15.9)	911 (17.3)
	Once/twice per month	83 (10.9)	367 (11.8)	249 (11.4)	699 (11.6)	56 (9.7)	326 (11.9)	233 (11.9)	615 (11.7)
	Once/twice per week	213 (28.0)	1002 (32.3)	759 (34.7)	1974 (32.6)	121 (21.0)	644 (23.5)	457 (23.4)	1222 (23.2)
	>Once/twice per week	194 (25.5)	915 (29.5)	686 (31.3)	1795 (29.7)	150 (26.0)	916 (33.5)	742 (37.9)	1808 (34.3)

^1^AS: Anticipate survival

Table 3 shows the distribution of health behaviour change variables overall and by anticipated survival at wave 1. Few participants changed their smoking behaviour over time, but quitting was more common that taking up smoking. Around half of participants changed their physical activity behaviour, with around one third become less active. Just over half of participants changed their alcohol consumption, with one around one quarter consuming less alcohol at wave 5 than wave 1, and around one fifth consumed more.

**Table 3:** Distribution of change in health behaviours between waves 1 and 5 by anticipated survival at wave 1, English Longitudinal Study of Ageing.

Behaviour	Category	Low AS^1^, n(%)	Moderate AS, n(%)	High AS, n(%)	Total, n(%)
Cigarette smoking	Change to less healthy	14 (1.8)	29 (0.9)	24 (1.1)	67 (1.1)
	No change	698 (91.7)	2864 (92.4)	2041 (93.3)	5603 (92.6)
	Change to more healthy	49 (6.4)	206 (6.7)	123 (5.6)	378 (6.3)
Physical activity	Change to less healthy	319 (42.0)	1021 (33.1)	720 (33.0)	2060 (34.2)
	No change	344 (45.3)	1501 (48.6)	1057 (48.4)	2902 (48.1)
	Change to more healthy	97 (12.8)	564 (18.3)	405 (18.6)	1066 (17.7)
Alcohol consumption	Change to less healthy	102 (17.7)	500 (18.3)	398 (20.3)	1000 (19.0)
	No change	293 (50.9)	1547 (56.5)	1085 (55.4)	2925 (55.5)
	Change to more healthy	181 (31.4)	689 (25.2)	474 (24.2)	1344 (25.5)

^1^AS: Anticipate survival

### Is greater anticipated survival cross-sectionally associated with healthier behaviours?

Ordinal logistic regression models of health behaviours by anticipated survival at wave 1, before and after adjustment for potential confounding variables are shown in Table 4. In fully adjusted models, those reporting low anticipated survival were more likely to be smokers than those reporting moderate anticipated survival; and those reporting high anticipated survival were less likely to be smokers. There was no difference in physical activity levels between those with low and moderate anticipated survival; but those reporting high anticipated survival had higher activity levels than those reporting moderate. In fully adjusted analyses, there was no evidence that alcohol consumption differed by anticipated survival.

**Table 4:** Ordinal logistic regression models of health behaviours by anticipated survival at wave 1, English Longitudinal Study of Ageing.

Behaviour	Anticipated survival	Unadjusted, OR^1^ (95% CI^2^)	Adjusted^3^, OR (95% CI)
Cigarette smoking (n = 6001)	Low	1.12 (0.92 to 1.38)	1.45 (1.15 to 1.83)
	Moderate	Reference	Reference
	High	0.74 (0.63 to 0.86)	0.75 (0.64 to 0.88)
Physical activity (n = 5995)	Low	0.49 (0.42 to 0.57)	0.95 (0.80 to 1.12)
	Moderate	Reference	Reference
	High	1.43 (1.29 to 1.59)	1.13 (1.01 to 1.26)
Alcohol consumption (n = 6000)	Low	0.71 (0.61 to 0.82)	0.91 (0.78 to 1.06)
	Moderate	Reference	Reference
	High	1.14 (1.04 to 1.26)	1.02 (0.92 to 1.13)

^1^ OR, odds ratio;

^2^CI, confidence intervals;

^3^adjusted for sex, age group, marital status, activities of daily living category, instrumental activities of daily living category, Centre for Epidemiologic Studies Depression Scale category and Index of Multiple Deprivation category

*Note*. Higher scores for health behaviours indicated ‘more’ of each behaviour (i.e. smoking, consuming alcohol more frequently, or being more physically active).

The hypothesis that greater anticipated survival has a cross-sectional association with healthier patterns of behaviour was confirmed for smoking, partially confirmed for physical activity, but not confirmed for alcohol consumption.

### Does greater anticipated survival increase the probability that behaviours will become ‘healthier’ and decrease the probability that behaviours will become ‘less healthy’ over time?

Table 5 shows unadjusted and adjusted relative risk ratios of behaviour change variables by anticipated survival categories. In fully adjusted analyses, non-smokers at baseline reported low anticipated survival were more likely to become smokers by wave 5, than those reporting moderate anticipated survival. No other differences in change in smoking behaviour by anticipated survival were found.

**Table 5:** Multinomial logistic regression of change in healthy behaviour between waves 1 and 5 by anticipated survival, English Longitudinal Study of Ageing.

		Adjusted for baseline behaviour,^1^ RRR^2^ (95% CI^3^)		Fully adjusted,^4^ RRR (95% CI)	
Behaviour	Anticipated survival	Change to less healthy versus no change	Change to more healthy versus no change	Change to less healthy versus no change	Change to more healthy versus no change
Cigarette smoking (n = 5998)	Low	2.05 (1.08 to 3.90)	0.81 (0.55 to 1.19)	2.38 (1.19 to 4.76)	0.90 (0.59 to 1.36)
	Moderate	Reference	Reference	Reference	Reference
	High	1.07 (0.62 to 1.85)	1.16 (0.87 to 1.55)	1.26 (0.71 to 2.22)	1.13 (0.83 to 1.52)
Physical activity (n = 5978)	Low	1.90 (1.57 to 2.28)	0.46 (0.36 to 0.60)	1.00 (0.81 to 1.23)	0.70 (0.53 to 0.94)
	Moderate	Reference	Reference	Reference	Reference
	High	0.86 (0.75 to 0.98)	1.24 (1.06 to 1.46)	1.05 (0.91 to 1.21)	1.08 (0.91 to 1.28)
Alcohol consumption (n = 5229)	Low	1.07 (0.82 to 1.40)	1.50 (1.20 to 1.86)	1.23 (0.92 to 1.63)	1.21 (0.96 to 1.54)
	Moderate	Reference	Reference	Reference	Reference
	High	1.18 (1.00 to 1.39)	1.00 (0.86 to 1.16)	1.15 (0.97 to 1.36)	1.11 (0.95 to 1.29)

^1^adjusted for relevant health behaviour at wave1;

^2^RRR, relative risk ratio;

^3^CI, confidence intervals;

^4^adjusted for sex, age group, marital status, activities of daily living category, instrumental activities of daily living category, Centre for Epidemiologic Studies Depression Scale category and Index of Multiple Deprivation category

Those reporting low anticipated survival were less likely to increase their physical activity levels between wave 1 and 5, than those reporting moderate anticipated survival in fully adjusted analyses. No other differences in change in physical activity by anticipated survival were found.

No statistically significant differences in change in alcohol consumption by anticipated survival were found in fully adjusted analyses.

The hypothesis that greater anticipated survival increases the probability that behaviours will become ‘healthier’ and decreases the probability that behaviours will become ‘less healthy’ at follow up was partially supported for smoking and physical activity, but not for alcohol consumption.

## Discussion

### Summary of findings

This is the first analysis we are aware of exploring cross-sectional and longitudinal relationships between anticipated survival (which we used as a marker of subjective perceptions of uncontrollable threats to health) and health behaviours. Our hypotheses were partially confirmed, though associations were inconsistent across behaviours.

As hypothesised, greater anticipated survival had a cross-sectional association with lower likelihood of smoking and higher physical activity levels. However, anticipated survival did not have a cross-sectional association with alcohol consumption.

As hypothesised, lower anticipated survival was associated with increased probability of taking up smoking and decreased probability of adopting healthier patterns of physical activity. However, there was no association between anticipated survival and change in alcohol consumption.

### Strengths and limitations of methods

Data from a large, population-based cohort study was used. This increases the representativeness and generalisability of the findings. It also allowed consideration of both cross-sectional and longitudinal relationships. Together these represent significant methodological improvements on the only previous work we are aware of on this topic.[12] Given the distinct possibility of reverse causation in this context, longitudinal data were particularly valuable. However, we can not exclude the possibility that intention to change (or not change) behaviour in the future influenced both baseline anticipated survival, and change in behaviours over time. Thus, whilst our longitudinal analyses provide stronger evidence on direction of causation than our cross-sectional analyses, the possibility of reverse causation can not be entirely excluded.

Despite the use of a large cohort, some behaviour changes were rare (particularly in relation to smoking) leaving small numbers in some cells (see Table 3). This was particularly noticeable in the older, less populated, age groups and exacerbated by the large number of predictors in our fully adjusted models. This means that some of our analyses may have been underpowered. Larger cohorts would be required to overcome this problem.

The measures of health behaviours used were self-reported throughout. This is likely to result in error, reducing our ability to detect differences where these may exist. Whilst responses to these questions may also be biased, it is likely that any such bias is consistent over time, making the longitudinal analyses less susceptible to misclassification than the cross-sectional analyses.

The variable target age that participants were asked to consider in the question used to determine anticipated survival means that like may not have been compared with like across participants. To explore this, the analyses were repeated additionally controlling for the difference between target age and age at data collection. This had no impact on the findings.

It is not clear that anticipated survival, as measured here, is necessarily an accurate measure of perceived uncontrollable threats to health. We used this variable as it was included in the ELSA dataset and allowed us to exploit this population-representative data source. Other authors have asked participants to estimate their anticipated survival if they were to make both maximal and minimal efforts to protect their health and using the difference between these as an estimate of uncontrollable threats to health.[12] Our measure of anticipated survival is likely to capture the impact of any intention to change health behaviours in the future, as well as uncontrollable threats to health. It may, therefore, underestimate true uncontrollable threats to health. If the degree of underestimation varies by socio-demographic variables, this could introduce bias. Although previous work suggests that anticipated survival correlates well with actual survival in this cohort,[21] further qualitative work is required to explore how individuals arrive at their judgements of anticipated survival and inform the development of accurate quantitative measures of uncontrollable threats to health.

A wide range of potential confounding variables were included in the analyses. However, as with any epidemiological analysis, the possibility of uncontrolled confounding cannot be excluded.

### Comparison of findings to previous research and interpretation of findings

Although some support was found for both of the hypotheses, support was not consistent across all health behaviours. In particular, effects in relation to alcohol consumption were absent.

The lack of statistically significant findings in relation to smoking, in particular, may indicate a lack of statistical power. Only 16% of the sample smoked at baseline and only 7% changed their smoking behaviour between waves 1 and 5 (see Table 2). In contrast 44% of participants changed frequency of alcohol consumption and 52% changed frequency of physical activity levels between waves 1 and 5. These differences may reflect the larger number of alcohol consumption and physical activity categories available—such that small changes in these behaviours, but not smoking, could be identified—as well as the relative difficulty of changing different behaviours.

Those reporting low anticipated survival were less likely to change to healthier patterns of physical activity and more likely to adopt smoking than those reporting moderate anticipated survival. It is possible that these relationships reflect uncontrolled confounding by poor health status. For example, those in poor health at baseline, who are likely to report poorer anticipated survival, may have had mobility limitations that restricted their ability to become more active. As we adjusted for a wide range of health variables at baseline we believe this is unlikely—but the possibility can not be excluded.

The finding that there were no relationship between anticipated survival and alcohol consumption may reflect the more complex association between alcohol and health than between both smoking and physical activity health, as well as public understanding of these relationships. In general less smoking and more physical activity is associated with better health, however there is a ‘j-shaped’ relationship between alcohol consumption and health outcomes with both high and low alcohol consumption appearing to be associated with poorer outcomes.[22] In addition, higher than recommended alcohol consumption tends to be viewed as normal and acceptable in the UK.[23, 24] In contrast, the majority of the population recognise smoking as harmful to health[8] and physical activity as beneficial.[25] The shape of the relationship between alcohol consumption and health, and the public’s understand of this, is, therefore, more complex than those between both smoking and physical activity and health.

In the fully adjusted longitudinal analyses, those reporting low anticipated survival showed some differences in behaviour change compared to those reporting moderate anticipated survival, but no differences were found between those reporting moderate and high anticipated survival. We also found that reporting low versus moderate anticipated survival was the most important predictor of true survival (rather than high versus moderate).[21] There appears to be something particularly predictive of death about reporting low anticipated survival that may influence both motivation and ability to pursue healthy behaviours.

### Implications of findings for research and practice

The findings reported here may be particular to older populations and it would be valuable to replicate them in cohorts reflecting the full age spectrum. Focusing on groups of individuals at high ‘risk’ of behaviour change (e.g. those taking part in specific behaviour change interventions) may also provide more statistical power. Future work should also explore how individuals arrive at their assessment of their anticipated survival, what factors they take into account when considering anticipated survival, and whether this is a good marker of perceived uncontrollable threats to health.

Our results suggest that individual assessments of uncontrollable threats to health may be important determinants of change in some health behaviours, particularly smoking and physical activity. Previous work suggests that the assessments of anticipated survival that we used as a proxy of uncontrollable threats to health tend to be accurate.[21] As such, individually-focused behaviour change interventions are unlikely to be able to address this particular determinant of health behaviours and health behaviour change. Wider, population-level, changes to systemic and societal factors, are likely to be necessary to change these variables. Achieving change in these factors requires consistent political will over the long term.

## Conclusion

In a representative cohort of older English adults, greater anticipated survival was cross-sectionally association with lower likelihood of smoking and higher physical activity levels. However, anticipated survival did not have a cross-sectional association with alcohol consumption. Over eight years of follow up, lower anticipated survival was associated with increased probability of taking up smoking, and decreased probability of adopting healthier patterns of physical activity. There was no longitudinal association between anticipated survival and alcohol consumption.
